# APSIC revised guidelines for prevention of central line associated bloodstream infections (CLABSI): a summary and position statement

**DOI:** 10.1017/ash.2026.10298

**Published:** 2026-02-03

**Authors:** Moi Lin Ling, Anucha Apisarnthanarak, Patricia Ching, Yee-Chun Chen, Glenys Harrington, Jin-Won Huh, Namita Jaggi, Keita Morikane, Sahadol Poonyathawon

**Affiliations:** 1 Infection Prevention and Epidemiology, Singapore General Hospital, Singapore, Singapore; 2 Thammasat University Hospital, Thailand; 3 The University of Hong Kong School of Public Health, Hong Kong; 4 National Taiwan University Hospital, Taiwan; 5 Infection Control Consultancy, Australia; 6 University of Ulsan College of Medicine, Republic of Korea; 7 Artemis Hospitals, India; 8 Yamagata University Hospital, Japan; 9 Chulalongkorn University Faculty of Medicine, Thailand

## Abstract

**Objective:**

To describe the revised Asia Pacific Society of Infection Control (APSIC) Guidelines for Prevention of Central Line Associated Blood Stream Infections (CLABSI) 2024.

**Design:**

The revised guidelines was developed by infection prevention and control experts and key opinion leaders from Asia Pacific.

**Setting:**

Emphasis on practical implementation of Central Line Insertion and Maintenance Bundles using quality improvement approach is recommended towards the goal of achieving zero CLABSI in any healthcare setting.

**Patients or participants:**

Any patients with a central line in a healthcare setting

**Interventions:**

Literature search was done for recent international updates in CLABSI prevention. Recommendations were evaluated for practical and feasible implementation in low resourced settings.

**Results:**

The key recommendations are listed in the APSIC CLABSI insertion and maintenance guidelines. Additional measures are recommended for use where CLABSI rates remain high despite implementation of all preventive strategies to achieve institutional goals.

**Conclusions:**

A surveillance program is recommended to monitor outcomes and compliance with bundles. Ongoing review of the performance data with appropriate interventions made should facilitate efforts towards zero CLABSI rate.

## Introduction

Use of central lines, or central vascular catheters (CVC) is common in both inpatient and outpatient settings (including long term care facilities and home care). Six studies done in Southeast Asian ICUs showed a high pooled incidence density of 4.7 per 1,000 catheter days (95% CI, 2.9–6.5; I2 = 83.8; χ2(5) = 30.9; *P* = .000) for central line associated blood stream infections (CLABSI).^1^ Approximately half of all CLABSIs are considered preventable when evidence-based guidelines are implemented for the insertion and maintenance of CVCs.^2^ Collaborative implementation of evidence-based interventions with stakeholder engagement, communication of opportunities for improvement and engaging local champions with feedback can reduce CLABSI.

APSIC convened experts in infection prevention and control as well as key opinion leaders from Asia Pacific region to review the APSIC Guidelines for Prevention of CLABSI published in 2015. The members of this workgroup are the authors of this paper.

The full revised APSIC Guidelines for Prevention of CLABSI is available at https://apsic-apac.org

## Methods

The workgroup reviewed previously published relevant international guidelines and recommendations and performed literature searches using PubMed. The workgroup met twice as well as discussed via email correspondence to complete the revision of the guidelines. Criteria used for grading the strength of recommendations and quality of evidence are described in Table 1.

**Table 1.** Categories for recommendation

Categories for strength of each recommendation	
**Category**	**Definition**
A	Good evidence to support a recommendation for use.
B	Moderate evidence to support a recommendation for use.
C	Insufficient evidence to support a recommendation for or against use.
D	Moderate evidence to support a recommendation against use.
E	Good evidence to support a recommendation against use.
**Categories for quality of evidence on which recommendations are made**	
**Grade**	**Definition**
I	Evidence from at least one properly randomized, controlled trial.
II	Evidence from at least one well-designed clinical trial without randomization, from cohort or case-controlled analytic studies, preferably from more than one center, from multiple time series, or from dramatic results in uncontrolled experiments.
III	Evidence from opinions of respected authorities on the basis of clinical experience, descriptive studies, or reports of expert committees.

The draft guideline was sent to an external reviewer, APSIC executive committee and national infection prevention and control societies who are member societies of APSIC in Asia Pacific for comments and feedback. A final draft was made following revision according to comments and feedback received. It was endorsed by the APSIC executive committee before upload at the APSIC website.

## Results

### CLABSI prevention bundles^3–10^

To prevent CLABSI, emphasis is placed on two aspects of central venous catheters: management: insertion and maintenance. CLABSI prevention bundles are created by combining proven evidence-based best practice interventions for patients with central venous catheters, both during insertion and maintenance. The central line insertion bundle comprises:

- Optimal site insertion
- Hand hygiene
- Alcohol-based chlorhexidine skin preparation
- Maximal barrier precautions
- Chlorhexidine-containing dressings

#### Recommendations

1. *Recommendations for central venous catheters*
  - *Weigh the risk and benefits of placing a central venous device at a recommended site to reduce infectious complications against the risk for mechanical complications (eg, pneumothorax, subclavian artery puncture, subclavian vein laceration, subclavian vein stenosis, hemothorax, thrombosis, air embolism, and catheter misplacement). [IA]*
  - *Avoid using the femoral vein for central venous access in adult patients when the catheter is placed under planned and controlled circumstances. [IA]*
  - *In adult intensive care unit (ICU) setting, subclavian vein catheter insertion is preferred to reduce infectious complications [IA]*
  - *In adult non-ICU settings, upper body insertion site is preferred to reduce infectious complications and thrombotic complications relative to a femoral insertion site [IIA]*
  - *Use ultrasound guidance for internal jugular catheter insertion [IA]*
  - *No recommendation can be made for a preferred site of insertion to minimize infection risk for a tunneled CVC. [Unresolved issue]*
  - *Place catheters used for hemodialysis and pheresis in a jugular or femoral vein, rather than a subclavian vein, to avoid venous stenosis. [IA]*
  - *Use of ultrasound guidance to place central venous catheters is recommended to improve first insertion-attempt success rates, reduce the number of cannulation attempts and insertion-related mechanical complications. [IB]*
2. *Perform hand hygiene procedures, either by washing hands with liquid soap and water or using alcohol-based hand rubs. Hand hygiene should be performed before and after palpating catheter insertion sites as well as before and after inserting, replacing, accessing, repairing, or dressing a central venous catheter. Palpation of the insertion site should not be performed after the application of antiseptic, unless aseptic technique is maintained. [IB]*
3. *Sterile gloves should be worn for the insertion of arterial, central, and midline catheters. [IA]*
4. *Use new sterile gloves before handling a new catheter when guidewire exchanges are performed. [IIA]*
5. *Prepare and clean the skin site with an alcoholic chlorhexidine solution containing a concentration of CHG greater than 0.5% or a 2% chlorhexidine-based preparation before central venous catheter insertion and during dressing changes. If there is a contraindication to chlorhexidine, tincture of iodine, an iodophor, or 70% alcohol can be used as alternatives. [IA]*
6. *Before accessing catheter hubs or injection ports, clean with an alcoholic chlorhexidine preparation or 70% alcohol to reduce contamination. [IIB]*
7. *Use Maximal Sterile Barrier precautions. [IB]*
8. *Use sterile chlorhexidine-containing dressings to cover catheter insertion site in patients over 2 months of age. [IA]*
9. *If moisture or oozing blood occurs at insertion site, apply sterile gauze/pad with transparent dressing until moisture or oozing blood are resolved and then apply sterile medication-impregnated semipermeable dressing (preferably chlorhexidine gluconate) as soon as possible. [IIB]*
10. *Healthcare facilities should have a procedure confirmation process (ie, CVC insertion checklist) documented during CVC insertion to ensure best practice and infection prevention process adherence. [IB]*

CLABSI maintenance bundle components include

- Daily review of line necessity and removal or replacement (if clinically indicated)
- Hand hygiene
- Disinfection of hubs before accessing and/or changing access hubs/lumens/devices.
- Aseptic dressing change technique.
- Change chlorhexidine impregnated dressing at least every 7 days.
- Standardize administration sets changes.

#### Recommendations

1. *Designated trained personnel who have demonstrated competence in insertion and maintenance of central venous catheters. [IA]*
2. *Promptly remove any central venous catheter that is no longer essential. [IA]*
3. *When adherence to aseptic technique cannot be ensured (ie, catheters inserted during a medical emergency), replace the catheter as soon as possible, that is within 48 hours. [IB]*
4. *Do not routinely replace CVCs, PICCs, hemodialysis catheters, or pulmonary artery catheters to prevent catheter-related infections. [IB]*
5. *Perform hand hygiene and wear either clean or sterile gloves when changing the dressing on central venous catheters. [IC]*
6. *If antiseptic barrier caps are used on needleless connectors, these must be changed after each access. [IIB]*
7. *Minimize contamination risk by scrubbing the access port/hubs with 70% alcohol or a chlorhexidine/alcohol preparation (≥0.5% CHG w/v in 70% alcohol) for at least 5 secs and accessing the port only with sterile devices. [IA]*
8. *Use either sterile gauze or sterile, transparent, semipermeable dressing to cover the catheter site. (IA)*
9. *Change chlorhexidine impregnated dressing at least every 7 days or according to the manufacturer’s instructions. [IA]*
10. *In patients not receiving blood, blood products or lipid formulations, replace administration sets that are continuously used, including secondary sets and add-on devices, up to but at least every 7 days. (IA)*
11. *Replace tubing used to administer propofol infusions every 6 or 12 hours, when the vial is changed, per the manufacturer’s recommendation. (IA)*
12. *Healthcare facility should use CVC maintenance checklist to document healthcare workers compliance with infection prevention best practices for patient safety and quality care. [IB]*

## Implementation

### Checklists^11^

We encourage the use of checklists to help healthcare workers comply with the components in each bundle. Compliance is associated with better patient outcomes, reduced infection rates, as well as lowering related healthcare costs.

### Culture^12–16^

A culture of targeting zero healthcare associated infections and zero tolerance for unsafe practices is characterized by the following:

- Setting the theoretical goal of elimination of CLABSI;
- An expectation that infection prevention and control (IPC) measures will be applied consistently by all healthcare workers, 100% of the time;
- A safe environment for healthcare workers to pursue 100% adherence, where they are empowered to hold each other accountable for infection prevention;
- Systems and administrative support that provide the foundation to successfully perform IPC measures;
- Transparency and continuous learning where mistakes and/or poor systems and processes can be openly discussed without fear of penalty;
- Prompt investigation of CLABSI of greatest concern to the organization and/or community; and
- Focus on providing real time data to frontline staff for the purpose of driving improvements.

#### Recommendations

1. *Hospital leadership and policymakers are to continue providing support to build a culture of zero tolerance. [IIIB]*
2. *Lines of accountability need to be established to link everyone in a hospital—from the board to frontline staff—so that everyone has a shared understanding of the organizational goals, their role in meeting these, goals and receives feedback (such as dashboards) on how they are performing. [IIIB]*

### Education and training^17–22^

All healthcare personnel involved with the insertion and maintenance care of CVCs should receive educational programs that address knowledge, critical thinking, behavior and psychomotor skills, and attitudes and beliefs of CLABSI prevention. Educate healthcare personnel regarding the indications for central venous catheter use and appropriate infection control measures to prevent central venous catheter-related infections.

#### Recommendations

1. *There should be focus on skill development and competency assessment in the organization. [IIIB]*
2. *The educational programs should be assessed for their content, relevance, staff knowledge, improvement and impact on work performance. [IIIB]*

### Cost-effectiveness analysis^20–22^

The core purpose of a cost evaluation for CLABSI is to provide a relative value to different healthcare interventions and to relate the value of the impact of these interventions to the value of specific health outcomes.

#### Recommendation

1. *Cost effective analysis to justify investment of resources to the IPC program. [IIIB]*

### Common barriers and feasible solutions to successful implementation^23^

Common barriers to implement best practices to reduce CLABSI include barriers at the organizational level (eg, the lack of leadership support and commitment, lack of a safety culture, lack of available resources), barriers at the unit level (eg, nurse staffing variables, such as inadequate nurse-to-patient staffing rations and use of non-permanent staff), barriers at staff level (eg, education, training, experience, and competency of staff). All of which can affect patient safety in several ways. To successfully implement CLABSI prevention program, healthcare workers need to understand the barriers to successful implementation of CLABSI prevention program in their institutions and try to overcome those barriers.

#### Recommendation

1. *Identifying and removing barriers to adherence to IPC practices is essential to successful implementation of best practices. [IIIB]*

### Additional strategies to reduce CLABSI^24–31^

Additional strategies to reduce CLABSI could be used in units within hospitals where CLABSI rates remain high despite implementation of all preventive strategies to achieve institutional goals. These include:

- Antiseptic daily bathing/wiping bath
- Antimicrobial and antiseptic impregnated catheters
- Antibiotic locks for long term central venous catheter usage
- Securement of central venous catheters
- Safety connectors and needleless system
- Antiseptic containing hub/connector cap/port protector

### Recommendations

1. *Antiseptic daily bathing or wiping bath has been shown to decrease CLABSI in the ICU patients >2 months of age. [IA]*
2. *Minocycline-rifampin or chlorhexidine-silver sulfadiazine impregnated catheters should be considered in adult patients whose catheter dwell time is expected to be >7 days in units where the CLABSI rate does not meet the set goal, and the prevention bundle of CLABSI has been implemented with a good compliance. [IA]*
3. *Patients using minocycline-rifampin or chlorhexidine-silver sulfadiazine-impregnated catheters should be monitored for side effects, such as anaphylaxis. [IIIB]*
4. *Prophylactic antimicrobial or antiseptic lock solution should be considered for the following:*
  - *Patients with long-term hemodialysis catheters [IA]*
  - *Patients with limited venous access and a history of recurrent CLABSI [IIB]*
  - *Pediatric cancer patients with long-term catheters [IB]*
5. *Use a suture less securement device to reduce the risk of infection for central venous catheters. [IIC]*
6. *Use of a split septum valve is preferred over some mechanical valves due to increased risk of infection with the mechanical valves [IIB]*
7. *Scrubbing the access port of connectors with an appropriate antiseptic (chlorhexidine, povidone iodine, an iodophor, or 70% alcohol) and accessing the port only with sterile devices. [IA]*
8. *Ensure that all needleless components are compatible to reduce the risk of leaks and breaks in the system. [IIB]*
9. *Change needleless components at least as frequently as the administration set, that is up to every 7 days. [IIB]*
10. *Change the needleless connectors no more frequently than every 72 hours, or according to the manufacturer’s recommendations. [IIB]*
11. *Use a needleless system to access IV tubing. [IC]*
12. *Use an antiseptic-containing hub/connector cap/port protector to cover connectors (IIB).*
13. *Use povidone iodine antiseptic ointment or bacitracin/gramicidin/polymyxin B ointment at the hemodialysis catheter exit site after catheter insertion and at the end of each dialysis session only if this ointment does not interact with the material of the hemodialysis catheter per manufacturer’s recommendation. [IB]*
14. *Mupirocin ointment should not be applied to the catheter insertion site due to the risks of facilitating mupirocin resistance and potential damage to polyurethane catheters. [IB]*

## Discussion

CLABSI is a preventable healthcare association infection. We recommend hospitals in Asia Pacific to implement a CLABSI prevention program facility-wide.^32^ A surveillance program is recommended to monitor outcomes and compliance with CLABSI prevention bundles. Ongoing review of the performance data with appropriate interventions made should facilitate efforts towards zero CLABSI rate.
